# Mitigating Gambling-Related Harms in Children and Young People: A Scoping Review of Interventions

**DOI:** 10.1007/s10899-025-10387-x

**Published:** 2025-05-19

**Authors:** Christina Davis, Kevin Davidson, Emily Arden-Close, Elvira Bolat, Constantina Panourgia

**Affiliations:** 1 University of the West of England, Bristol, UK; 2 Goldsmiths College, University of London, London, UK; 3 Bournemouth University, Fern Barrow, Poole, BH12 5BB UK

**Keywords:** Children, Young people, Gambling-related harms, Intervention, Effectiveness, Scoping review

## Abstract

Gambling among children and young people (CYP) has been associated with a range of adverse outcomes, including financial consequences, psychological distress, social difficulties, and increased risk of experiencing gambling-related harms in adulthood. Given the growing accessibility of gambling activities through digitally- enabled experiences and the expanding regulatory concerns, there is a pressing need for evidence-based interventions to mitigate harm in this vulnerable population. Despite numerous interventions aimed at reducing gambling-related harm among CYP, systematic analysis of their effectiveness remains limited. To address this gap, we conducted a comprehensive scoping review of literature published between 2008 and 2025, using the Preferred Reporting Items for Systematic Reviews and Meta-Analyses (PRISMA) extension for scoping reviews and the Arksey and O’Malley five-stage protocol. The PAGER Framework was employed to critique the literature. We searched eight electronic databases, supplemented by manual reference checks, for peer-reviewed studies on interventions to prevent or reduce gambling-related harm in CYP up to age 25. Of 11,972 articles identified, 40 met the inclusion criteria. Our analysis revealed four key themes: effective interventions often integrate multiple methods and actively engage participants, are theory-driven and delivered by trained practitioners, are tailored to developmental needs, and incorporate digital technologies. Common issues across studies included the absence of follow-up assessments and the use of adult questionnaires with CYP. We recommend addressing the social context and environment rather than focusing solely on individual behaviour, involving CYP in intervention development, evaluating long-term effects, and considering underserved populations such as CYP with special educational needs. This review underscores the urgent need for rigorously evaluated, evidence-based interventions to reduce gambling-related harm in CYP across educational, healthcare, and youth work settings.

**Supplementary Information:**

The online version contains supplementary material available at 10.1007/s10899-025-10387-x.

Gambling, recognised as a significant public health issue with severe consequences for individuals and families (Tulloch et al., 2022), has increasingly become a normalised recreational activity (Bjørseth et al., 2021). Evidence from various countries indicates that individuals often begin to participate in gambling activities between the ages of 10 and 19 years (Petro, 2013). In recent years, gambling prevalence has surged in the UK, notably among children and young people (CYP), with 27% of those aged 11–17 having engaged in gambling activities (Gambling Commission, 2024), despite the legal gambling age being 18 (Gambling Act, 2005). This trend is partially attributed to the convergence of gaming and gambling, leading to the rise of digital platforms that simulate gambling experiences without formal classification as gambling, often appealing to younger audiences (Delfabbro et al., 2022; Kim & King, 2020; Zendle & Bowden-Jones, 2019).

CYP’s interaction with digital technologies significantly increases their exposure to gambling advertisements and services, especially through social media platforms (Pitt et al., 2023; Smith et al., 2019). These advertisements often employ strategies and celebrities intended to appeal to young audiences (Pitt et al., 2018; Rossi & Nairn, 2021), portraying gambling as socially desirable and enjoyable (O’ Loughlin & Blaszczynski, 2018). The allure of “easy money” presented in these promotions can be particularly enticing to CYP (Sklar & Derevensky, 2011). Given their limited understanding of probabilities and reduced control over outcomes, they may not fully grasp the complexities of gambling and its marketing, leading to misconceptions about betting odds and expected outcomes (Pitt et al., 2016; Rossi & Nairn, 2021). Moreover, CYP have a natural inclination towards sensation-seeking and risky behaviours (Arnett, 2000; Steinberg et al., 2018; Worthy et al., 2010), which increases their susceptibility to gambling-related harms (Blakemore & Choudhury, 2006; Chambers & Potenza, 2003; Griffiths & Parke, 2010; Kräplin & Goudriaan, 2019; Rogers et al., 2019).

Various interventional strategies have been proposed for mitigating gambling-related harm, which encompass public education campaigns, psychological treatment for those at risk, family-based support, implementation of restrictive features on gambling machines, and constraints on gambling advertising (Rogers et al., 2019). McMahon et al.’s (2019) umbrella review of systematic reviews on gambling harm interventions reveals a predominance of evaluations of individual-level harm and demand-reduction interventions,^1^ indicating a paucity of research on supply-reduction^2^ and contextual interventions^3^. Recognising the importance of systems-level thinking, some reviews underscore the need for public health approaches and interdisciplinary programs in preventing gambling-related harms (Blank et al., 2021; Kourgiantakis et al., 2016). However, most such interventions have focused on adults.

Notably, evidence from systematic reviews suggests that interventions tailored specifically to children and young people (CYP), including preventive programmes, are limited (Giménez Lozano & Morales Rodríguez, 2022; Grande-Gosende et al., 2020; Keen et al., 2019; Kourgiantakis et al., 2016; Ladouceur et al., 2013; Monreal-Bartolomé et al., 2023; Oh et al., 2017). Concerns have been raised about the lack of evidence regarding the longitudinal impact of school-based education programs on gambling behaviour, due to insufficient inclusion of long-term follow-up measures (Ladouceur et al., 2013). Additionally, reviews highlight the absence of family-focused prevention strategies targeting at-risk groups, such as children with parents experiencing gambling problems (Kourgiantakis et al., 2016). Overall, in the dynamic context of evolving technology, comprehensive evidence regarding the array of intervention strategies tailored for CYP at risk of gambling-related harm is lacking (Monreal-Bartolomé et al., 2023).

This scoping review aims to address two primary objectives: firstly, to evaluate the spectrum of existing interventions targeting CYP at risk of gambling-related harm, and secondly, to identify how effective these interventions are in supporting CYP in relation to gambling. We aimed to identify areas lacking sufficient research and thus meriting further investigation. Furthermore, this scoping review aimed to provide valuable insights to a diverse audience, encompassing policymakers, practitioners, and public health organisations, as suggested by Peters et al. (2020).

## Method

This scoping review adhered to the Preferred Reporting Items for Systematic Reviews and Meta-Analyses (PRISMA) extension for scoping reviews as outlined by Tricco et al. (2018). It followed Arksey and O’Malley’s (2005) comprehensive five-stage protocol. To ensure transparency and reproducibility, the review followed a protocol, which was registered with the Open Science Framework (OSF) and is accessible via the following link: 10.17605/OSF.IO/MYHZF. To ensure thorough description and critical analysis of included literature, we employed the PAGER Framework (Bradbury-Jones et al., 2021). This approach both augmented the descriptive and evaluative aspects of our review and strengthened the methodological foundation provided by Arksey and O’Malley (2005).

### Stage 1: Review Questions

We aimed to answer the following research questions:

1. What is the evidence related to existing services/interventions for CYP experiencing gambling-related harms nationally and internationally?
2. How effective are existing interventions in preventing and reducing gambling and gambling-related harms amongst CYP?

### Stage 2: Search Strategy

**Fig. 1:**
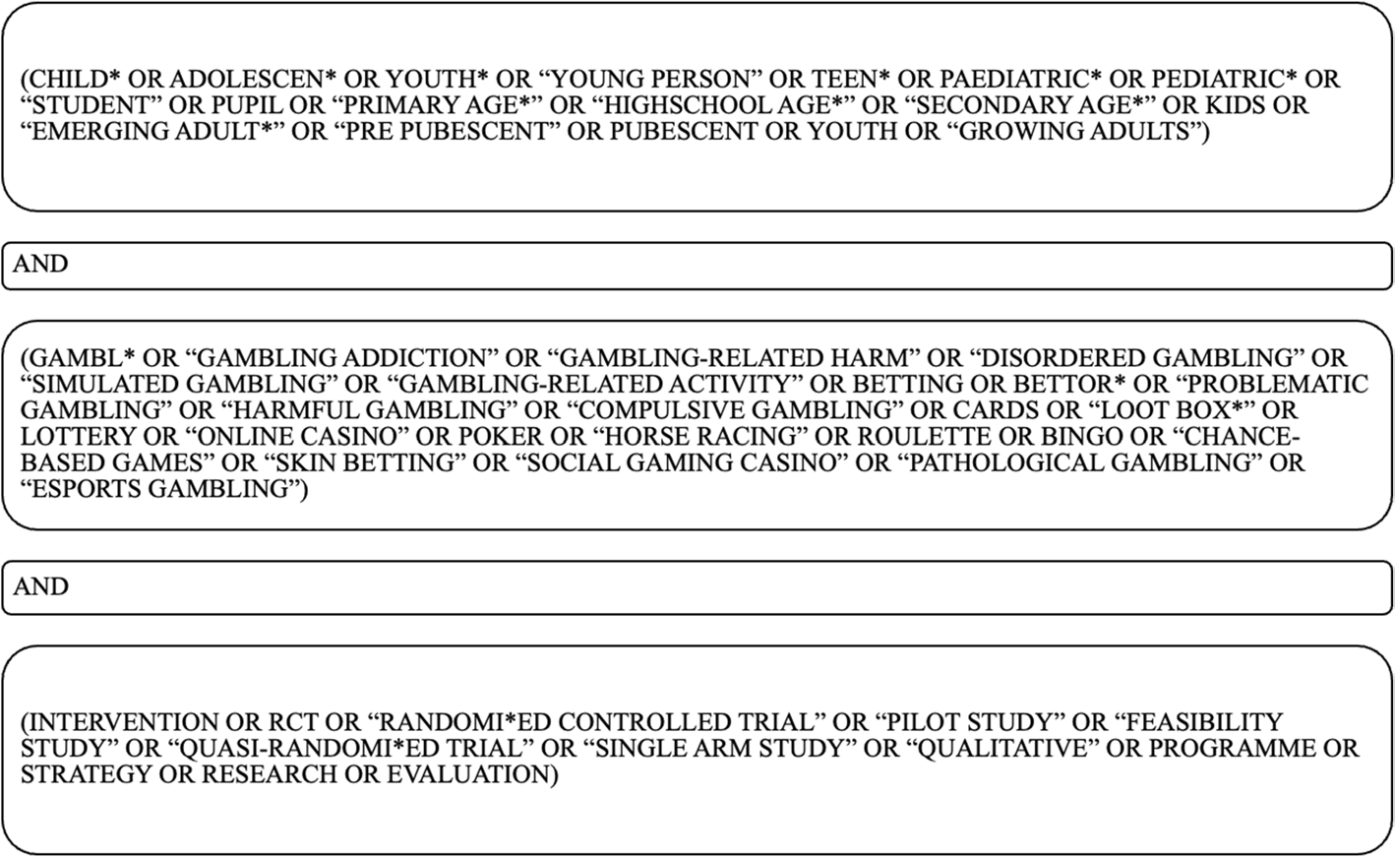
List of search terms

We defined the search terms by first identifying keywords and phrases to conduct a targeted search (Peters et al., 2020). From 30th April 2023 to 16th March 2025, we searched the databases CINAHL + , APAPsycINFO, The Cochrane Library, Web of Science, Medline and SCOPUS to ensure interdisciplinary coverage. The search terms (Fig. 1) included a combination of keywords for the concepts of gambling, child or adolescent, and intervention, combined with the Boolean operator “AND.”

The search strategy was formulated through the research group’s expertise, in collaboration with professionals who had work experience with children and/or in gambling (Experts by Experience Group), including a young people’s service manager at a gambling prevention charity, a drama therapist, a family coach with children’s social services, a head of personal, social, health and economic education and citizenship in a secondary school, and a mentor working with disadvantaged people from Black, Asian, Minority Ethnic and Refugee backgrounds. We utilised the dictionary definition of gambling as: *“the betting or staking of something of value, with consciousness of risk and hope of gain, on the outcome of a game, a contest, or an uncertain event whose result may be determined by chance or accident or have an unexpected result by reason of the bettor’s miscalculation.”* (Encyclopaedia Britannica, 2023).

Overall, 11, 952 articles were identified through database searching. An additional 20 articles were found through hand searching reference lists (total *n* = 11,972). Duplicates (*N* = 9,346) were removed, leaving 2,626 studies for title and abstract screening. The full text of 88 articles were screened. After discussion, 48 studies were excluded, leaving 40 studies in the final analysis.

### Stage 3: Selection of Studies

Inclusion and exclusion criteria were developed by the research team (see Table 1). Recognising that most existing interventions to mitigate gambling-related harm primarily focus on adults — likely reflecting the legal gambling age of 18 in the UK and similar jurisdictions—our criteria included studies involving young people up to 25 years old. This expansion aimed to capture vulnerable demographics such as university students and “emerging adults”. Conversely, interventions designed for adult populations but also available to individuals under 25 were excluded. For instance, interventions specifically targeting university students were included, whereas those primarily intended for adults but also accessible to university students were excluded. To reflect the significant technological advancements in the gambling industry, notably the widespread adoption of smartphones since 2007, we limited inclusion to English language articles published after 2008. Studies from regions with gambling laws markedly different from those in the UK, including countries in Asia and Africa where gambling is broadly illegal, were excluded to maintain relevance to our context.

**Table 1:** Inclusion and exclusion criteria

Inclusion Criteria	Exclusion Criteria
● Studies on children and young people up to 25 years ● Interventions for children and young people experiencing gambling-related harm ● All peer-reviewed primary research study designs ● Studies in English language	● Studies involving participants aged over 25 years ● Studies conducted before 2008 ● Countries that are not comparable to the UK (e.g., countries where gambling is illegal for all ages, not just children aged under 18 years)

**Fig. 2:**
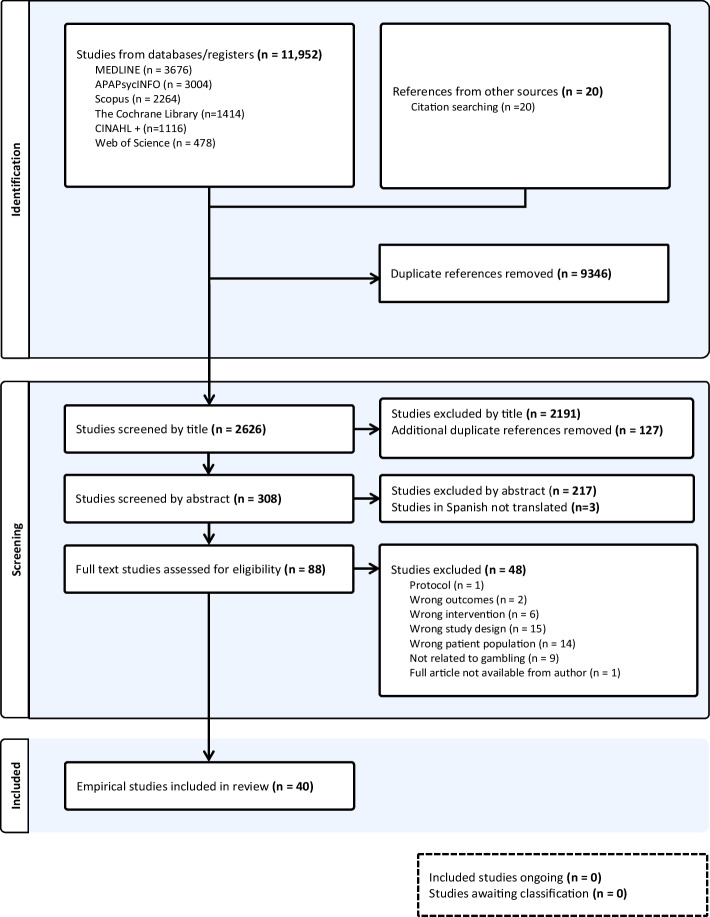
Prisma diagram for the interventions, practices and systems to support Children and Young People (CYP) at risk of gambling harm study

Studies were exported to Covidence for primary screening of the results. The PRISMA flowchart is presented in Fig. 2. This process was managed through the EndNote Research tool, at which point duplicates were identified and deleted. Two members of the research team (CD and KD), independently screened abstracts, using the inclusion and exclusion criteria to select studies for full text review. CD and KD then independently screened full texts, with any discrepancies being resolved by a third member (EAC).

### Stage 4: Charting Data

To identify key concepts, information from included articles was charted using an Excel spreadsheet. Data extracted from the selected articles included author, date, and title; study location, population, sample size and context; study design, duration and comparator (control group – if applicable); intervention type, strategic approach and theoretical basis; and outcomes, conclusions and future recommendations. Data was extracted by two members of our research team (CD and KD) and ratified by co-authors who reviewed 20% of the extracted papers at random.

During data extraction, it became important to identify important categories and characteristics of interventions so patterns within the data would be more easily observable. As part of this process, it was also necessary to streamline terminology because some studies used different terminology for the same interventional approach. Categories which may require additional explanation are summarised in Table 2.

**Table 2:** Categories and characteristics of interventions

Categories	Types (if applicable)	Detailed Overview
***Prevention Strategy***	*Universal*	*‘Universal’ interventions are aimed at all youth (*Ladouceur et al., 2013*)*
	*Indicated*	*‘Indicated’ interventions target CYP who display noticeable psychological or behavioural signs of problem gambling behaviour but do not meet the diagnostic criteria for gambling disorder, usually assessed *via* screening (*Dickson et al., 2004*)*
	*Selective*	*‘Selective’ interventions target CYP who share a characteristic which is known to increase risk of gambling-related harm such as coming from a single-parent household or living in an area of high crime rates or low socioeconomic status (*Dickson et al., 2004*)*
	*Treatment*	*‘Treatment’ interventions target CYP with a diagnosable gambling disorder, such as Disordered Gambling, according to the DSM-5 criteria (*André et al., 2022*)*
***Type of intervention***	*Psychological interventions*	*‘Psychological’ interventions encompassed well-established forms of psychological therapy such as CBT or ACT*
	*Educational interventions*	*‘Educational’ interventions were designed to increase knowledge about how gambling works, gambling distortions and the risks of problematic gambling behaviour*
	*Psychoeducational interventions*	*‘Psychoeducational’ interventions (sometimes also referred to as education* + *skills) were designed to increase awareness of psychological skills in relation to gambling such as coping strategies and addictive behaviours*
	*Social norms approaches*	*‘Social norms approaches’ employed personalised normative feedback*
	*Public health initiatives*	*‘Public health initiatives’ focused on altering the environment in which risks of gambling harms emerge, such as changing the legal age for gambling or removing slot machines from specific environments*
	*Harm minimisation interventions*	*‘Harm minimisation’ interventions employed pop-up messages to flag risks of gambling harm during a gambling experience*
***Type of gambling targeted***		*Some interventions targeted specific types of gambling, such as slot machines, casinos or sports betting, whereas other interventions targeted gambling behaviour in general*
***Mode of delivery***	*In-person*	*‘In-person’ describes interventions delivered by a practitioner in the same physical space as an individual young person or group of young people*
	*Interactive screen-based*	*‘Interactive screen-based’ described interventions accessed through a device or screen necessitating the young person’s active engagement. These covered apps, web-based games or video games*
	*Didactic screen-based*	*‘Didactic screen-based’ involved passive engagement through electronic devices, encompassing formats like PowerPoint presentation, video or docudrama*
***Age range of study participants***	*Young/emerging adults*	*18–25 years old*
	*Adolescents*	*13–18 years old*
	*Children*	*Up to 13 years old*

Study quality was appraised using the Critical Appraisal Skills Programme (CASP) checklists, which provide a suite of tools designed to systematically evaluate the trustworthiness, relevance, and outcomes of published research. We used the following checklists (details can be found in supplementary materials), CASP Randomised Controlled Trial Checklist to appraise studies publishing randomised controlled trials (RCTs)—20 studies, CASP Cohort Study Checklist to appraise studies publishing cohort research—seven studies, and a combination of checklists to evaluate remaining studies that employed diverse methodological approaches—13 studies. All CASP checklists invite reviewers to rate studies using a ‘yes/no/can’t tell’ measure in relation to different aspects of validity, results, and clinical relevance. Quality was individually assessed by two members of our research team (CD and KD).Comparison between the researchers revealed high inter-rater reliability.

### Stage 5: Collating, Summarising and Reporting Results

The results from peer-reviewed papers were grouped and summarised according to the elements from the PAGER framework: patterns, advances, gaps, evidence for practice and research recommendations (Bradbury-Jones et al., 2021). The data was presented in tabular form and then described narratively, synthesising information about approaches and findings across studies. The Experts by Experience Group reviewed the draft version and final report and provided feedback during an online meeting and via email.

## Results

Forty studies were included in the review. Detailed information describing the included studies is reported in Table 3.

**Table 3:** Characteristics of included studies

Study No	Author and Year	Country	Participants	Comparison	Measures	Prevention-type /strategic approach/theoretical basis (where mentioned)	Intervention Type	Intervention Mode of Delivery, Provider, Intensity and Duration	Outcomes
1	Grahler et al. (2024)	Germany	Intervention group: 2367 vocational students, and 1458 (61.6%; M age 19.0, SD 3.5 years; 830/1458, 56.9% male) of them provided full data. Of these 1458 students, 894 (61.3%) started a challenge. Thus, 894 data records with baseline, follow-up, and app data could be included in the analysis The least chosen challenge was gambling with 31 (4.2%) students	None- focus only on the Intervention Group	Frequency of app use (in days); challenge choice; personal relevance of challenge selection; congruent use; Dichotomous outcomes (change vs no change) referred to past-month substance use, gambling, and media-related behaviours	Indicated (Cognitive Behavioural Theories)	Psychological	Mode of delivery: smartphone app; Provider: app-based, web-based; Intensity and duration: 2-week intervention followed by a 30-day follow-up period	15/31 participants who chose the gambling challenge chose the challenge congruent to their personal relevance. Congruent use was associated with increased odds of health-promoting change. For gambling, this was an Odds Ratio of 2.07, (95% CI 1.21–3.55; P = .008) Additionally, the more problematic consumption habits a student had, the more likely he or she was to have a health-promoting change (OR 2.43, 95% CI 1.99–2.98; p = < .001) However, frequent use showed lower odds for health-promoting change for gambling (OR 0.47, 95% CI 0.26–0.86; P = .009) Main findings: 1)61.3% of the students used the app and therefore the intervention 2)Almost half of the users used the app in congruence with personal relevance suggesting high awareness of personal habitual behaviours 3) Health-promoting changes were influenced by congruent use, choice of challenge, and personal relevance score, possibly providing empirical support for effectiveness of the app
2	Pietsch et al. (2023)	Germany	1458 college students; mean age 19.0; (31 in gambling arm); 53.9% male; 12.27% of full sample (12.69% of intervention arm) had a 30-day prevalence of gambling	1403; no treatment but access to app at end of study	Substance-related behaviour in the last 30 days (assessed dichotomously with yes/no); plus, assessment of other healthy and risky behaviours	Indicated (Cognitive Behavioural Theories)	Psychological	*Mode of Delivery:* smartphone; *Provider:* researcher; *Intensity and duration:* 14 days	Overall improvement in health-related behaviour. Odds Ratio for gambling reduction was 1.14 (0.81–1.61) in favour of intervention group (use of intervention decreased risk of gambling by 14%)
3	Choliz et al. (2022)	Spain	2372; 14–19 years-old; 51.2% male; recruited from Spanish High schools	None (quantitative pre–post intervention data)	Gambling patterns and gambling disorder survey; DSM-IV	Universal Reno Model (Blaszczynski et al., 2004)	Educational	*Mode of delivery:* face-to-face; *Provider:* psychologist specialising in gambling and addiction; *Intensity and duration:* 2 sessions	Reduction in gambling for traditional and online gamblers; gambling problems reduced with both at risk and gambling disorder. However, females- reduction in at-risk but NOT gambling disorder (possibly low % of females); younger children benefited most; older youth- no reduction in gambling disorder (only at risk)
4	Donati et al. (2022)	Italy	*n* = 900 (short term effects) 56% males, mean age 14.58 years; long term effects *n* = 662 (58% males, mean age 15.57 years); 12%—at-risk gamblers and 6% problem gamblers (PG) G; 56–58%/ 61% male	None	Gambling-related Knowledge scale -for adolescents; Random events knowledge test- youth version; non-gambling task; Superstitious Thinking Scale; Gambling Expectancies Questionnaire; Gambling Related Cognitions Scale -Revised for Adolescents; Gambling Task; Gambling Behaviour scale for adolescents; DSM-V gambling disorder	Universal (Dual Process Theory)	Educational	*Mode of delivery:* face-to-face; *Provider:* teachers; *Intensity and duration:* 7 months	Gambling-related erroneous thoughts reduced; Short-term: Increase in cognitive protective factors and reduction in affective risk factors; Long-term: decrease of gambling frequency and reduction in gambling problem severity
5	André et al. (2022)	Sweden	9 participants aged 13–17 years: 33%—“addicted gamblers”; 78%—“disordered gamers”; 89% of all participants—male	None (pilot study)	GASA (gaming screening for adolescents); CLiP (adult problem gambling screen); self-report questions	Treatment (Cognitive Behavioural Theories + RP)	Psychological	*Mode of Delivery:* face-to-face*; Provider:* psychiatrists and psychologists with training in CBT; *Intensity and duration:* 7 sessions of 45 min over 7 weeks	RP efficacy on gambling: 2/3 (problem gamblers) did not gamble after treatment. 2/6 (who did not gamble before treatment) endorsed gambling after treatment. 1/3 gambled before and after treatment. Participant's evaluation: 5/9 participants participated in evaluation reporting qualitatively
6	Latvala et al. (2022)	Finland	524,914 young people aged 14–16 years-old: 50% male	None	Survey data asking about gambling frequency (School Health Promotion Study)	Universal	Public health initiatives / Environmental interventions	Government legislation	Adolescent Gambling significantly decreased over time. It appears that raising the legal gambling age had a permanent effect on under-age gambling. However, differences in gambling by adolescents’ family SES increased during the study period, indicating widening inequalities in gambling among adolescents
7	Primi & Donati, (2022)	Italy	72 students aged 15–19 years; High-risk: 89% male; high-levels of socio-economic inequality; high-prevalence of related risk-behaviour (cannabis and alcohol-use); poor school achievement; high proportion of immigrants. 75% gamblers, 16% At-risk problem gamblers ( ARPG)	*n* = 50 assessment only	Gambling-related Cognitions Scale- Revised for Adolescents (GRCS-RA); Illusion of Control; Predictive Control and Interpretative bias; Gambling Behaviour Scale for Adolescents (GBS-A)	Selective (Dual Process Theory)	Educational	*Mode of delivery:* face-to-face*; Provider:* intervention provider trained about this model; *Intensity and duration:* A single 2-h session given twice in 2 weeks	Significant reduction in cognitive distortions related to gambling
8	Tani et al. (2021)	Italy	393 students aged 13–19; 84% male; 34 ARPG; 19 problem gamblers (student group)	No training group *n* = 174 students, 18 teachers. Assessment only	South Oaks Gambling Screen Revised for Adolescents (SOGS-RA); Gambling Related Cognitions Scale; Gambling Attitudes Scale	Universal (Extended Parallel Process Model (EPPM); Action Research Model)	Educational	*Mode of delivery:* face-to-face; *Provider:* trained teachers; *Intensity and duration:* 4 modules × 4 h per module	Reduction of some cognitive distortions and misconceptions related to the economic profitability of gambling in the intervention group; only the group that used the trained teachers reduced gambling behaviour in students
9	Dodig Hundric et al. (2021)	Croatia	629 young people aged 14–17-year-old; 6.7% High severity of adverse psychosocial consequences; 13.4% low-moderate severity of adverse psychosocial consequences	None	Gambling Behaviour; Gambling related knowledge; gambling related cognitive distortions; Problem solving skills; resisting peer-pressure skills; general self-efficacy; problem gambling severity scale	Universal (Dickson’s Model of Youth Gambling-Dickson et al., 2002)	Psychoeducational	*Mode of delivery:* face-to-face *Provider:* trained school psychosocial professionals (school counsellors) and teachers; *Intensity and duration:* 9 workshops over 9 weeks (45 min per session); a 2-h interactive lecture with parents and 2-h interactive lecture with staff	Significant improvement in knowledge, illusion of control; no significant impact on socioemotional skills
10	McAfee et al. (2020)	USA	255 students; 62% male; 76.5% white; mean score of 4.47 on CPGI (Canadian Problem Gambling Index)	86 in PFB-TXT (Personalised Feedback-Text); 73 in PFB-EDU (Personalised Feedback- Education); *n* = 96 assessment-only control	South Oaks Gambling Screen (SOGS); Brief Biosocial Gambling Screen (BBGS); gambling timeline followback; Canadian Gambling Problem Index; Gambling Norms; gambling-related cognitive distortions; gambling protective behavioural strategies	Selective (Personalised Normative Feedback)	Social norms approaches	*Mode of delivery:* smartphone*; Intensity and duration:* text messages over 28 days	PFB condition had no direct effect relative to control condition on dependent variable
11	Calado et al. (2020)	Portugal	111 students aged 11–18 years; 46% male; 16% ARPG (for total group; 21.4% ARPG in experimental group)	*n* = 55 students, normal school activity	Misconceptions and knowledge of gambling questionnaire (Ferland et al., 2002); DSM-IV-J-MR; Attitudes toward Gambling Scale (Wardle et al., 2011); Brief Sensation-seeking scale (Hoyle et al., 2002)	Universal (Cognitive Behaviour Theories)	Educational	*Mode of delivery*: face-to-face; *Provider:* authors of intervention; *Intensity and duration:* 5 didactic units over 5 weeks lasting 1 h	Short-term effects: experimental group demonstrated significant effects for knowledge about gambling, misconceptions, attitudes and total hours spend gambling. Long-term effects in experimental group only (n = 39): No change between post-test and follow-up results suggesting permanence of change for 6 weeks; non-significant interactions for gambling frequency, amount of money spent gambling and sensation seeking. Also- percentage of ARPG inside the experimental group from pre-test to follow-up decreased from 21.4% to 7.7% at follow-up
12	Ren et al. (2019)	USA	16,262 students aged 8–18.; 21% received the intervention more than once; prevalence of PG = 9.4%	None	Modified South Oaks Gambling Screen for Teens (MSOGST)	Universal (Dickson’s model of Youth Gamlbing -Dickson et al. (2002)	Educational	*Mode of delivery:* face-to-face, video-based, printed publication; *Provider:* schools; *Intensity and duration:* multiple sessions of 45–60 min over 5 years	Students receiving multiple interventions had higher scores than those receiving single intervention. Gambling knowledge increased over time with multiple interventions but not with single intervention; prevalence of PG decreased among students receiving the intervention twice as compared to once. Effect not confirmed for 3 + times
13	Zhou et al. (2019)	Canada	122 mean age 23 years old; 75.41% female. No information on problem gambling	Two treatment (GameSense vs Control) and 3 Gambling Outcome Conditions (win, lose, break-even); *n* = 61- played the game but did not receive prevention programme	SOGS-RA; Gambling Cognitive Questionnaire; Attitude Towards Gambling Scale; Post-Gambling Questionnaire;	Universal	Psychoeducational	*Mode of delivery:* online game; *Intensity and duration:* 9 modules	Students in intervention had higher awareness about gambling fallacies and knowledge. Treatment participants reported had slightly fewer intentions to continue to gamble. Initial results suggest the programme decreased subjective likelihood of future wins (compared to control where winning increased estimates of future wins)
14	McGivern et al. (2019)	UK	45 university students aged 18 + . *n* = 31 non-problem gamblers; *n* = 9 low-level PG; *n* = 5 moderate level PG. overall mean PGSI score = 0.85	Control messages (press ok to continue). no information on sample size	Total wager amount	Selective	Harm minimisation strategies	*Mode of delivery:* online game; *Provider:* computer; *Intensity and duration:* 15 min play, fixed to lose	Expenditure-specific messages differed significantly from both generic warning messages and control with significantly lower wager amounts in both cases
15	Diehr et al. (2018)	USA	431 total (214 = casino message; 215 = sports betting message aged between 18–25 years; 49.2% male; 66.1% didn't gamble	None	Central Intercept Survey	Universal	Public health initiatives/ Health communication strategies	*Mode of delivery:* printed publication; *Provider:* public health communication; *Intensity and duration:* 1 week	Positive feedback on risk/reward considerations on messages, raising awareness of gambling disorders
16	Donati et al. (2018)	Italy	34 students aged 15–19 years old; 100% Male; 81% (*n* = 23)—non-problem gamblers; 12% (*n* = 4) ARPG; 6% (*n* = 2) PG	*n* = 18 assessment-only condition. normal school activity	Gambler's Fallacy Task (Primi & Chiesi, 2011 ); Superstitious thinking scale (Kokis et al., 2002); Gambling-Related Cognitions Scale (Raylu & Oei, 2004); South-Oaks Gambling Screen- Revised for Adolescents (SOGS_RA, Winters et al., 1993)	Selective (Dual Process Theory)	Educational	*Mode of delivery:* face-to-face; *Provider:* developmental psychologist expert in adolescent gambling research; *Intensity and duration:* 2-h session delivered once a week for 2 weeks	Experimental Group reported a reduction in cognitive distortions; Short-term; small possible changes in gambling frequency
17	Parham et al. (2019)	USA	73 students aged 11–18 years: disproportionately racial/ethnic minorities and low SES	None	Seven questions on the pre-test assessed students’ involvement in gambling activities: knowledge of gambling and chance	Selective	Psychoeducational	*Mode of delivery:* face-to-face; *Provider:* Master's-level school-based mental health clinicians, licensed /supervised outpatient therapists embedded within specific schools; *Intensity and duration:* 3 × 45 −60-min session	Significant increases in student awareness and knowledge following participation in MD-Smart Choices. Focus group data collected from program facilitators suggested high student engagement and participation, program feasibility, and ease of implementation. PG was not assessed
18	Broussard and Wulfert (2017)	USA	90 college students: 93% gamblers; 50% male	Handout unrelated to gambling—no specific sample size information	SOGS (South Oaks Gambling Scale); GBQ (Gamblers Beliefs Questionnaire)	Selective (biopsychosocial model of problem gambling and a behaviour-analytic perspective)	Psychological	*Mode of delivery:* computer, printed publication, slot machine; *Intensity and duration: S*ingle session	Exposure to the accelerator or educational handout decreased judgements on probability of winning; accelerator condition played fewer trials om the slot machine than controls
19	Huic et al. (2017)	Croatia	190 young people aged 14–17 years old; 15% ARPG in intervention group; 14% ARPG in control; 67.6% male	*n* = 101 assessment only condition	Knowledge about gambling/betting; Cognitive Beliefs scale (Ricijaš et al., 2011); Problem-solving skills; Resistance to peer-pressure skills; Generalised Self-Efficacy Scale; Gambling Activities; Problem Gambling (Canadian Adolescent Gambling Inventory, Tremblay et al., 2010)	Universal (Dickson's Model of Youth Gambling -Dickson, et al., 2002)	Psychoeducational	*Mode of delivery:* face-to-face; *Provider:* 2 experts in adolescent gambling and school interventions; *Intensity and duration:* 6 units of 90 min each	Risk factors (knowledge and cognitive distortions)—reduced in intervention group; Protective factors- no evidence for effect; No significant behaviour change
20	St-Pierre et al. (2017)	Canada	280 students in total; 13–16 years-old	*n* = 139 (69 males) control condition with no video intervention; preventive intervention condition (*n* = 141; 71 males)	Gambling Attitudes Scale; Gambling Injunctive Norms Scale; Perceived Control over gambling refusal scale; NAE's for gambling; Gambling Intention Scale; Gambling Activities Questionnaire	Universal (Theory of Planned Behaviour)	Educational	*Mode of delivery:* video-based; *Intensity and duration:* 25-min video with booster session 1 week later	Control and intervention groups had a small significant increase in positive gambling attitudes and positive peer and family subjective norms (unintended negative consequences); the video was not effective in producing changes in behaviour
21	Canale et al. (2016)	Italy	168 14–18-year-olds; 58% male; 8.3% problem gamblers	*n* = 73 received personalised feedback without intervention	South Oaks Gambling Screen–Revised for Adolescents (SOGS-RA); Gambling Attitude Scale (GAS)	Selective and indicated (Cognitive Behavioural Theories + Motivational Interviewing)	Psychological	*Mode of delivery:* computer*; Provider:* web-based; *Intensity and Duration:* 4 weeks (1 session per week)	No change in gambling frequency, expenditure or attitudes; gambling problems significantly decreased in the intervention group
22	Dixon et al. (2016)	USA	18 college-aged (mean age: 18–19 years) disordered gamblers; all had a SOGS score of 3 or above; 100% male sample	A mixed-design study involving a 2 × 2x2 factorial design was conducted, with the factors being group (ACT, Control), condition (wins, losses), and time (pre, post). The study included nine participants performing an fMRI gambling task without any treatment intervention	fMRI -brain activation patterns; psychometric surveys: acceptance and action questionnaire II; valued living questionnaire; mindful awareness attitude scale	Indicated. (ACT and Relational Frame Theory)	Psychological	*Mode of delivery:* face-to-face; *Provider:* ACT therapist; *Intensity and Duration:* 8 h of ACT	Higher rates of psychological flexibility and mindfulness in the intervention group. Additionally, FMRI results reported participants in the Act intervention group showed greater brain activation patterns for winning spins when compared to the initial scanning session. Participants in the control group showed no differentiation in brain activity following winning spins
23	Nordmyr and Österman (2016)	Finland	10,000 participants aged 14–21 years (data for 15–17-year-olds)	None	Lie/Bet tool (Johnson et al., 1997)	Universal	Public health initiatives / Environmental interventions	Government legislation	A statistically significant decrease in the frequency of problem gambling was found with 18–19-year-olds between 2011–2014 (aged 15–16 years in 2011 and directly affected by legislation change)
24	Neighbors et al. (2015)	USA	252 students mean age 23; all scored 2 + on SOGS; 59.5% male	*n* = 128 attention-control feedback	South Oaks Gambling Screen (SOGS); Gambling Quantity and Perceived Norms scale; Gambling Problems Index; measure of identification with groups	Selective (Personalised Normative Feedback; Social Identity Theory)	Social norms approaches	*Mode of delivery:* printed publication*; Intensity and duration:* Single session	Significant intervention effects in reducing perceived norms for quantities lost and won; Reduction of gambling problems at the 3-month follow-up. All intervention effects except reduced gambling problems remained at the 6-month follow-up. Intervention effects were moderated by self-identification with other student gamblers, suggesting that PNF worked better at reducing gambling for those who more strongly identified with other student gamblers
25	Martens et al. (2015)	USA	Students mean age—21; all scored 3 + on SOGS or 1 + on Brief Biosocial Gambling Screen; 58–62% male; mean SOGS score = 4.77	*n* = 109 assessment-only control—no informational materials; *n* = 111 PFB; *n* = 113 EDU	South Oaks Gambling Screen (SOGS); Brief Biosocial Gambling Screen (BBGS); Gambling Timeline Followback; Canadian Problem Gambling Index	Selective (Personalised Normative Feedback)	Social norms approaches	*Mode of delivery:* printed publication*; Intensity and duration:* single session	Participants in PFB condition reported fewer dollars gambled and fewer gambling-related problems than those in AOC. There were no differences between the PFB and the EDU conditions
26	Raisamo et al. (2015)	Finland	8101 young people aged 12–16 years-old	None	S elf-reported 6-month prevalence of slot machine use overall and by venue	Universal	Public health initiatives / Environmental interventions	Government legislation	6-month prevalence of slot-machine use among 12–16-year-olds declined from 44% in 2011 to 13% in 2013 (significant)
27	Donati et al. (2014)	Italy	181 students aged 15–18 years; 64% male; 41% ARPG in intervention condition	*n* = 36 usual school activity	South Oaks Gambling Screen-Revised for Adolescents (SOGS-RA); Questionnaire of Attitudes and Knowledge About Gambling; Gambler’s Fallacy Task (GFT); Gambling Attitude Scale (GAS); Superstitious Thinking Scale (STS)	Selective	Educational	*Mode of delivery:* face-to-face, video-based*; Provider:* developmental psychologist expert in adolescent gambling and school intervention*; Intensity and duration:* 2 × 1 h	Improvements in correct knowledge of gambling and reducing misconceptions; reduction in perception of probability and superstitious thinking. however, no effects in at risk/problem gamblers; the percentage of at risk/problem gamblers decreased from 41–28% from pre-test to follow-up
28	Celio and Lisman (2014)	USA	136 college students who reported participation in at least one gambling activity (e.g., card gambling, skill games, sports gambling, etc.) during the past 30 days; 55% male; mean age 19 years	*n* = 68 attention- control feedback unrelated to gambling; *n* = 68 PNF condition	Balloon Analogue Risk Task (BART); Measure of Gambling task (Pick a Card; PAC test); Gambling Quantity and Perceived Norms Scale	Selective ( Personalised Normative Feedback)	Social norms approaches	*Mode of delivery:* printed publication; *Intensity and duration:* single session	Marked decrease in perception of other student's gambling and lower risk-taking performance in the intervention condition
29	Rossow et al. (2013)	Norway	3,855 students aged 13–18 years	None	School Survey, contributing towards 1) LieBet score 1 + ; 2) LieBet score 2.; 3) SOGS-RA 2 + ; 4) SOGS-RA 4 + ; 5) Self-perceived gambling problem	Universal	Public health initiatives/ environmental interventions	Government legislation	A small proportion reported they had changed their gambling behaviour
30	Walther et al. (2013)	Germany	2109 young people aged 10–15 years old: 50.4% male, no reported problem gamblers	*n* = 1221 assessment only	Lifetime gambling; gambling frequency; gambling attitudes and beliefs scale; gambling knowledge	Universal	Psychoeducational	*Provider:* trained teachers; *Intensity and duration:* single session 90 min (four-unit media education program, which contained one unit on gambling)	Increased gambling knowledge and decreased problematic gambling attitudes; decrease of current gambling but no significant influence on lifetime gambling
31	Wohl et al. (2013)	Canada	72 participants aged 18–28 years old; recreational gamblers (70% female) undergraduates; Problematic Gambling %- not given	Mixed 2 (group: animation vs neutral video) × 2 (pop-up reminder, pop-up no reminder)	Erroneous Cognitions ( informational Biases Scale, Jefferson & Nicki, 2003); Limit Detection; adherence to pre-set limit	Selective (Educational material vs pop-up messages)	Harm minimisation strategies	*Mode of delivery:* video-based; *Provider:* computer; *Intensity and duration:* 9-min animation and reminders	Experimental group who viewed educational animation) reported significantly less gambling-related erroneous cognition than those who viewed the neutral video. Additionally, those in the pop-up condition adhered to monetary limits more than control. However, there was no difference between the future gambling practices in the two groups (education and neutral) that received a pop-up reminder, suggesting that the reminder is more important than the educational material
32	Lupu and Lupu (2013)	Romania	75 participants aged 12–13 years: 48% male. Problem Gambling not assessed	*n* = 23 free discussions about subjects of interest	Gambling Knowledge Questionnaire	Universal (Rational Emotive Education; Cognitive Behavioural Theories)	Educational	*Mode of delivery:* face-to-face, computer*; Provider:* one psychologist and a psychiatrist + 3 psychology students; *Intensity and duration:* 10 weekly meetings of 50 min each	Significant difference in knowledge about gambling. Subjects in AC + REE condition obtained significantly more correct answers than REE alone
33	Todirita and Lupu (2013)	Romania	81 children aged 12–13 years; 45.7% male, Problem Gambling not assessed	*n* = 24 wait-list control; *n* = 29 information only; *n* = 28 Rational Emotive Therapy	Gambling knowledge; Illusion of control; attitudes and cognitive errors	Universal (Rational Emotive Education; Cognitive Behavioural Theories)	Educational	*Mode of delivery:* face-to-face, computer; *Provider:* one psychologist and one psychology student; *Intensity and duration:* 10 weekly meetings	Significant improvement in gambling knowledge in the information-only experimental condition; significant improvement in erroneous attitudes and cognitions, illusions of control and misconceptions in both the REE and Information-only condition. However, the Information-only condition appeared to have a greater impact on children's knowledge, attitudes and erroneous cognitions about gambling
34	Larimer et al. (2012)	USA	Individual PFI (personalised Feedback Intervention) = 52 2); Group CBI = 44; all participants met on average 2.25 DSM-IV PG criteria; 10.2% met 5 or more criteria; 65.3% male; all participants were taken from university	Assessment only control *n* = 51	SOGS; gambling quantity and perceived norms scale (GQPN); gambling problems index, DSM-IV; beliefs about control scale	Indicated (CBI + PFI/ Cognitive Behavioural Theories)	Psychological	*Mode of delivery:* face-to-face; *Provider:* trained therapist (treatment integrity monitored); *Intensity and Duration:* CBI 4–6 weeks, PFI: single session of 60–90 min	PFI displayed reduced perceptions of gambling frequency norms; CBI displayed reduced illusions of control; Results indicated reductions in both interventions for gambling consequences and DSM-IV criteria. PFI resulted in reductions in gambling frequency
35	Williams et al. (2010)	Canada	949 young people aged 14–20 years; according to the DSM-IV-MR-J, 3.2% of students were problem gamblers at baseline; according to the Self-Reported Problem Gambling measure, 5.2% of students were problem gamblers at baseline	Control group (*n* = 433 at baseline, 291 at follow-up). Received intervention afterwards (WLC)	Gambling attitudes; Gambling knowledge scale; Gambling fallacies scale; decision-making and problem-solving skills; participation in high-risk activities; DSM-IV-MR-J	Universal	Psychoeducational	*Mode of delivery*: face-to-face; *Provider:* one psychologist and one psychology student; *Intensity and duration:* 10 weekly meetings	Improvement in knowledge and negative attitudes and reduction in gambling fallacies; significant improvements in problem-solving and decision-making; reduction in frequency but no change in problem gambling or expenditure
36	Hansen and Rossow (2010)	Norway	20,695 participants in 2006	None	Gambling frequency and expenditure; SOGS-RA; Lie/Bet tool	Universal	Public health initiatives / Environmental interventions	Government legislation	Expenditures on slot machines as well as overall gambling frequency showed a substantial decrease after the intervention
37	Petry et al. (2009)	USA	117 college student problem and pathological gamblers: 78–90% male; mean age = 20 years old	*n* = 34 assessment only control; *n* = 32 brief advice; *n* = 30 motivational enhancement therapy; *n* = 21 MET and CBT	Addiction Severity Index (ASI); SOGS; DSM-1 V; timeline followback; treatment service review	Indicated (Motivational Enhancement Therapy + Cognitive Behavioural Theories)	Psychological	*Mode of delivery:* face-to-face*; Provider:* therapists; *Intensity and Duration:* 1–4 × 50 min session	Reduction in gambling to a greater extent in participants assigned to any brief intervention than those assigned to assessment-only control. MET- only condition displayed consistent beneficial effects when compared to the AOC- condition. MET + CBT had mixed results (possibly due to low attendance). Brief Advice was also seen to have significant beneficial effects on some outcome measures
38	Taylor and Hillyard (2009)	USA	8455 students aged 12–18 years; Most participants (60.3%) indicated that they did not have any problems with gambling (MSOGST score = 0), 29.7% reported “some” problems with gambling (MSOGST score = 1–4), and 10% had a score of 5 or higher, which represented “probable pathological gamblers”	None	Modified South Oaks Gambling Screen for Teens (MSOGST; Taylor, 2008)	Universal (Dickson's Model of Youth Gambling- Dickson et al., 2002)	Educational	*Mode of delivery:* face-to-face, CD-ROM*; Provider:* teachers; *Intensity and Duration:* 45 min + CD-ROM	The program was successful at increasing knowledge of gambling and the negative effects it can have, over the short term
39	Turner et al. (2008a)	Canada	374 students from grades 5–12 (10–18 years-old)	*n* = 162 assessment only condition; *n* = 212 in experimental condition	14-item version of random event knowledge test (REKT; Turner et al., 2008a, 2008b); the SOGS-RA (Winters et al., 1993); shortened version of the luck and skill questionnaire (Derevensky et al.,^1997^); gambling activities checklist; activities preference questionnaire (based on Allen et al., 1992, but changed to a rating scale), which ask students how much they enjoyed several different activities; open-ended questionnaire asking the students how they would cope with various stressful situations	Universal (Self-Efficacy Theory; Social Inoculation and Reasoned Action)	Psychoeducational	*Mode of delivery*: face-to-face; *Provider*: authors of paper / researchers + student actors; *Intensity and duration*: single 1-h session	Significant improvement in gambling misconceptions; no significant improvement of coping or problem-solving skills, gambling attitudes or behaviours; No effect on gambling behaviour
40	Turner et al. (2008b)	Canada	201 total participants, 15–18-year-olds; 3.5% with possible pathological gambling problem (score 4 + on SOGS-RA); 66/201 were male; 135/201 were female	*n* = 101 assessment only condition; 100 = intervention condition	South Oaks Gambling Screen-Revised for Adolescents (SOGS-RA); Preventative Resource Inventory (PRI); Random Events Knowledge test; Problem Gambling Awareness; assessment of student's retention to content	Universal (Self-Efficacy Theory; Social Inoculation and Reasoned Action)	Psychoeducational	*Mode of delivery:* face-to-face; *Provider*: teachers; *Intensity and duration:* 7 sessions	Significant improvement in problem gambling awareness; significant improvement in understanding of randomness and significant improvements to understanding self-monitoring, coping skills (especially among high-risk students)

### Study Location

Most academic studies we reviewed were conducted in North America (*n* = 12 in the USA and *n* = 6 in Canada). The remaining studies were conducted in Europe, as follows: Italy (*n* = 6), Finland (*n* = 3), Germany (*n* = 3), Croatia (*n* = 2), Romania (*n* = 2), Norway (*n* = 2), Spain (*n* = 1), Sweden (*n* = 1), Portugal (*n* = 1) and UK (*n* = 1).

### Sample Populations

Most studies (*n* = 40) recruited their sample populations from schools, colleges or universities. The remaining study (André et al., 2022) recruited participants from a child and adolescent psychiatry service in Sweden and screened for eligibility concerning disordered gaming and/or problem gambling. The cross-sectional studies drew data from school surveys. No studies were from sample populations that were actively seeking help.

Sample sizes ranged from nine participants for a pilot feasibility study (André et al., 2022) to 524,914 respondents in a cross-sectional study (Latvala et al., 2022), representing a total of approximately 607,304 young people between 2008 and 2025.

Most studies (*n* = 17) included more male than female participants, ranging from 55%−100% male samples. Thirteen studies had a roughly equal split between male and female participants, ranging from 45.7%−53.9% male participants. In three studies, gender split was not reported. Seven studies reported a higher proportion of females than males, ranging from 54%−75.4% female.

### Methodologies Employed

Of the 40 empirical studies, 20 employed Randomised Controlled Trials to test intervention efficacy (Broussard & Wulfert, 2017; Calado et al., 2020; Canale et al., 2016; Celio & Lisman, 2014; Dixon et al., 2016; Donati et al., 2014, 2018; Larimer et al., 2012; Lupu & Lupu, 2013; Martens et al., 2015; McAfee et al., 2020; Neighbors et al., 2015; Petry et al., 2009; Pietsch et al., 2023; Primi & Donati, 2022; St-Pierre et al., 2017; Todirita & Lupu, 2013; Turner et al., 2008a; Walther et al., 2013; Zhou et al., 2019). Five were repeated measures cross-sectional studies (Hansen & Rossow, 2010; Latvala et al., 2022; Nordmyr & Österman, 2016; Raisamo et al., 2015; Rossow et al., 2013). Six studies were experimental pre- and post-designs with one group (Chóliz et al., 2022; Dodig Hundric et al., 2021; Donati et al., 2022; Grahler et al., 2024; Parham et al., 2019; Taylor & Hillyard, 2009). Four studies were experimental pre- and post- designs with two groups (Huic et al., 2017; Turner et al., 2008b; Williams et al., 2010; Wohl et al., 2013). Two studies were longitudinal (Ren et al., 2019; Tani et al., 2021), two were pilot studies (with typically small sample sizes) (André et al., 2022; McGivern et al., 2019), and one an exploratory study (Diehr et al., 2018).

### Quality Analysis of Academic Studies

Studies were quality assessed using the CASP checklist (see Supplementary Materials for further details). All studies addressed a clearly focused issue, identifying potential and actual confounding factors in both design and analysis. Most studies demonstrated strong randomisation processes, blinding, and baseline similarity. Non-randomised studies generally reported their methodology clearly. However, seven RCTs and six non-randomised studies did not report dropout rates. While many interventions were delivered by trained professionals, none discussed integrity of the intervention in their results.

School-based RCTs often employed cluster-randomisation by class or school to minimise bias from intervention effects. However, when randomisation is by class, it is difficult to eliminate discussion of the intervention across classes with the control groups, potentially compromising the interventions’ integrity or results. Most studies reported results comprehensively, accurately and clearly. However, three studies did not report power calculations (Diehr et al., 2018; Taylor & Hillyard, 2009; Turner et al., 2008a).

The quality assessment acknowledged the heterogeneity of the populations studied. Studies were designed and evaluated across different cultural contexts (Italy, Croatia, USA) or for different age groups, meaning the clinical relevance cannot be determined universally. Additionally, some studies used measurement instruments specific to the individual study or for adult populations. Cohort studies examining legislation changes in specific countries may have limited relevance elsewhere. Furthermore, the low numbers of participants who reported engaging in gambling could affect the generalisability and impact of the results across different populations.

### Analysis of Studies by Intervention Type

Eight studies, consisting of seven interventions used psychological interventions (André et al., 2022; Broussard & Wulfert, 2017; Canale et al., 2016; Dixon et al., 2016; Grahler et al., 2024; Larimer et al., 2012; Petry et al., 2009; Pietsch et al., 2023). Four studies used a social norms approach such as personalised feedback (Celio & Lisman, 2014; Martens et al., 2015; McAfee et al., 2020; Neighbors et al., 2015) Two studies used a mixture of psychological and social norms approaches (Larimer et al., 2012; Petry et al., 2009) and one used psychological behavioural techniques combined with an educational handout (Broussard & Wulfert, 2017). Twelve studies, consisting of eight interventions, used an educational only prevention strategy (Calado et al., 2020; Chóliz et al., 2022; Donati et al., 2014, 2018; 2022 ; Lupu & Lupu, 2013; Primi & Donati, 2022; Ren et al., 2019; St-Pierre et al., 2017; Tani et al., 2021; Taylor & Hillyard, 2009; Todirita & Lupu, 2013).

Eight studies used a combination of educational and skills training, basing prevention programs on psychoeducational principles rather than solely building knowledge of gambling (Dodig Hundric et al., 2021; Huic et al., 2017; Parham et al., 2019; Turner et al., 2008a, 2008b; Walther et al., 2013; Williams et al., 2010; Zhou et al., 2019). These papers detailed seven distinct interventions.

Five studies detailed results from wider public health initiatives from two countries (Hansen & Rossow, 2010; Latvala et al., 2022; Nordmyr & Österman, 2016; Raisamo et al., 2015; Rossow et al., 2013). One study explored the acceptability of health communication posters (Diehr et al., 2018) and two studies employed a harm minimisation strategy (McGivern et al., 2019; Wohl et al., 2013).

The rationale for the studies varied depending on the type of intervention focused on. For example, psychological interventions such as Cognitive Behavioural Therapy (CBT), Motivational Interviewing (MI) or Acceptance and Commitment Therapy (ACT) were designed to change behaviour, hence were aimed at individuals who were already gambling or showed signs of gambling harm. Studies applying personalised feedback (social norms) interventions aimed to either correct misperceptions of gambling behaviour or reduce gambling behaviour. These interventions were aimed at college students who reported gambling but not necessarily experiencing gambling harms. Educational intervention studies were mainly based in school settings and aimed to correct misconceptions and increase accuracy of knowledge about gambling. Educational interventions also looked at changing behaviour; however, the samples were from large school populations where not all participants reported gambling. Psychoeducational approaches were also mainly school-based and less focused on behaviour change. These interventions were aimed at the widest age range and included the youngest participants (minimum age 10 years old). Public health initiatives were aimed at reducing the capacity to engage in physical gambling behaviour by restricting the environment. They formed a community approach that was less focused on changing misconceptions or improving skills. The last group of interventions were aimed at minimising gambling harms in individuals who reported currently gambling. These two studies were conducted with university students aged over 18 years old.

### Analysis by Target Behaviour

21 studies looked at general gambling behaviour (Canale et al., 2016; Celio & Lisman, 2014; Chóliz et al., 2022; Dodig Hundric et al., 2021; Donati et al., 2014; Huic et al., 2017; Larimer et al., 2012; Lupu & Lupu, 2013; Martens et al., 2015; McAfee et al., 2020; Neighbors et al., 2015; Parham et al., 2019; Petry et al., 2009; Primi & Donati, 2022; Ren et al., 2019; Rossow et al., 2013; St-Pierre et al., 2017; Tani et al., 2021; Todirita & Lupu, 2013; Walther et al., 2013; Williams et al., 2010; Zhou et al., 2019). Eleven looked at specific modes of gambling (Broussard & Wulfert, 2017; Diehr et al., 2018; Dixon et al., 2016; Hansen & Rossow, 2010; Latvala et al., 2022; McGivern et al., 2019; Nordmyr & Österman, 2016; Raisamo et al., 2015; Wohl et al., 2013) and four looked at gambling and cognitive distortions (Donati et al., 2018, 2022; Turner et al., 2008a, 2008b). Two studies examined gambling alongside other addictions, such as substance use, social media use, gambling and gaming (Grahler et al., 2024; Pietsch et al., 2023), one explored risky behaviours as well as gambling (Calado et al., 2020); and one investigated gambling and disordered gaming (André et al., 2022).

### Analysis of Outcome Measures Used

Over 40 different measures were used across the 40 studies. Outcome measures mainly related to the nature and focus of the intervention design. For example, psychological Interventions used outcomes such as the Mindful Attention Awareness Scale (Brewer et al., 2010 in Dixon et al., 2016) or the Beliefs about Control Scale (Moore & Ohtsuka, 1999, in Larimer et al., 2012). In contrast, psycho-educational interventions examined (among others) gambling knowledge, cognitive distortions, problem-solving skills, attitudes to gambling and self-efficacy. Educational interventions assessed cognitions such as the Gambling-Related Cognitions Scale—revised for adolescents (GRCS-RA; Raylu & Oei, 2004, in Primi & Donati, 2022), the Superstitious Thinking Scale (Kokis et al., 2002 in Donati et al., 2018), Gambler’s Fallacy Task (Primi & Chiesi, 2011 in Donati et al., 2018) and the Gambling Attitudes Scale (GAS, Delfabbro & Thrupp, 2003 in Donati et al., 2014). Social norms approaches used the Brief Biosocial Gambling Screen (BBGS, Gebauer et al., 2010 in McAfee et al., 2020), Perceived Norms Scales (Neighbors et al., 2002, in Neighbors et al., 2015) and measures of identification within groups.

However, by far the most used scales for both identification of gambling prevalence, and analysis of intervention outcomes, were the South Oaks Gambling Screen (SOGS; used 6/39 times) (Lesieur & Blume, 1987) or a variation of this such as the SOGS-RA (used 9/39 times) (Winters et al., 1993 or the Modified SOGS for Teens (used 2/39 times) (MSOGST; Taylor, 2008); and the DSM-IV criteria (used 3/39 times) (Wickwire et al., 2008), or the junior version of this, the DSM-IV-Multiple Response-Juvenile (used 2/39 times) (Fisher, 2000). High variance in the choice of screening and outcome measurement tools limits comparisons of results between studies. A lack of culturally and developmentally appropriate assessment tools required authors of interventions to use adult scales, youth scales adapted from adult scales in the past, or to devise novel outcome measurement tools.

### Thematic Analysis

Four themes, which were identified and examined using the PAGER framework (See Table 4) are discussed below.

**Table 4:** Overview of key results: PAGER framework

Themes	Patterns and Advances	Gaps	Evidence for Practice	Research Recommendations
*Effective interventions incorporate multiple methods and actively engage participants in diverse ways*	Interventions that combined activities aimed at enhancing knowledge, developing skills, and exploring associated risk factors were found to be more effective in raising awareness and addressing negative attitudes about gambling harms than those focusing on a single method or risk factor. Interventions that included an active, hands-on component also appeared to yield more effective results compared to those without such elements	Long-term follow-ups were not explored. Variability in study participants, design, and outcome measures	Research in other fields has identified several potential interventions. For instance, in the context of excessive digital use, interventions involving exercise (Tseng et al., 2022) and narrative therapy (Gong et al., 2022) have been reported as beneficial. Personality-targeted interventions show promise in addressing adolescent alcohol consumption (Conrod et al., 2011). Additionally, art-based interventions have been utilised in treating substance use addiction (Maina et al., 2022)	Creative interventions (Maina et al., 2022) and the application of student-friendly services—distinct from those designed for adults—should be considered. It's essential to address barriers to treatment, such as raising awareness, improving access to available treatments, enhancing self-management of conditions, and ensuring treatment accessibility. Special attention should be given to making interventions inclusive for minority groups and SEND (Special Educational Needs and Disabilities) populations, utilising creative solutions to meet their unique needs
*Effective interventions are theory-driven and implemented by trained practitioners*	Most studies included a clear theoretical basis for the design and development of the intervention models, and these theoretical foundations were diverse. Two Italian studies highlighted that training teachers to deliver support significantly reduced gambling behaviour and enhanced gambling knowledge among children (Donati et al., 2022; Tani et al., 2021)	There was often a lack of reporting on the actual reduction in gambling behaviour. Additionally, no analysis was conducted to determine which theoretical approach best suits the target population and behaviour. The long-term effects of trained practitioners as an outcome measure have also not been assessed in current studies	Evidence suggests that gambling can act as a 'safe space' for young people, allowing them to escape everyday stressors and difficulties (Gluck, 2021). This indicates that education-based interventions alone may not be sufficient	Consider exploring alternative theories on gambling behaviours among children and young people (CYP), as well as theories on effective intervention strategies. Interventions should be designed around these insights
*Effective interventions are developmentally appropriate, tailored to individual characteristics, relevant to the target population, and may incorporate support from family networks*	Studies have examined the cultural relevance of interventions (Parham et al., 2019). For younger participants (8–11 years old), interventions that took their developmental stages into account were often found to be effective	Research into the neurobiology and developmental stages of children highlights gaps in addressing help-seeking behaviours, particularly among younger demographics, with limited studies involving 10–11-year-olds. It is rare for young people (YP) to be consulted directly, despite the acknowledged risks of age-related gambling behaviours. This creates a contradiction when interventions fail to consider the disparity between emotional regulation, cognitive development, and educational practices. Consulting YP is crucial, as prevention tactics introduced at age 13 are often too late. Effective interventions should overcome barriers to treatment and consider specific needs, as certain groups are more vulnerable to gambling harms due to co-occurring issues. Evidence suggests that designing interventions requires thoughtful consideration of diverse factors, including the unique vulnerabilities of children with additional needs or those experiencing compounding harms. However, literature exploring effective interventions for children and young people (CYP) with SEND (Special Educational Needs and Disabilities) or other additional needs is limited. No studies explicitly included children and adolescents with SEND. Different motivations and gambling practices may require tailored interventions, particularly when co-morbid conditions such as anxiety, depression, learning difficulties, conduct disorders, or substance use are present	Research indicates that individuals with learning difficulties, ADHD, autism, and other groups, such as those with hearing loss, are more vulnerable to gambling-related harms than the general population (Breyer et al., 2009; Chamberlain et al., 2023; Faregh & Derevensky, 2011; Geidne et al., 2016; Taylor et al., 2015). Adolescence is a period marked by risk-taking behaviours, but it is crucial not to frame this developmental stage negatively or attribute blame to the individual or their age. Children and adolescents who lack adequate parental support are at higher risk of developing psychological and social issues (Ding & Li, 2023). Additionally, research suggests that interventions effective for younger children may not work as well for adolescents, highlighting the need for age-appropriate approaches (Yeager et al., 2018)	It is critical to conduct additional targeted research focused on children and young people (CYP) with SEND (Special Educational Needs and Disabilities). Family-based education and therapy may also be beneficial. Interventions should recognise that not all children attend school and should be designed to reach those who are not in traditional educational settings. It's important to consider broader social and environmental factors, as well as the unique aspects of childhood and adolescence, to ensure interventions are appropriately targeted
*Effective interventions incorporate engagement with digital technologies*	Technologies used in interventions included smartphones, online games, and a simulated gambling accelerator game. These studies reported positive behavioural changes in participants, such as increased awareness of gambling fallacies, improved knowledge, reduced intentions to gamble, and significant improvements in correcting erroneous attitudes, illusions of control, and misconceptions. There was also evidence of a reduction in gambling activities	The researchers of these studies did not explore why incorporating digital technologies proved effective, focusing instead on their suitability and benefits in terms of engagement, brevity, and convenience	Research on digital interventions with young people in other fields suggests that the effectiveness of apps depends on the user's personal understanding of the need for behaviour change and their motivation to seek treatment (Fitzgerald & McClelland, 2016; Liverpool et al., 2020). In the context of digital addiction, digital technologies have been explored with children as young as seven years old (Ding & Li, 2023). A recent review by Halldorsson et al. (2021) indicates that evidence supporting the use of digital interventions for children and young people (CYP) is emerging, particularly in addressing mental health problems	Further evidence is needed in both gambling and non-gambling contexts

#### Theme 1: Effective Interventions Include Multiple Methods and Involve Active Engagement of Participants

A 5-week intervention conducted in Portugal (Calado et al., 2020) aimed to educate and reduce sensation-seeking behaviours in adolescents, with a focus on harm from gambling and gaming. The intervention consisted of five one-hour didactic sessions, delivered weekly during school hours, which involved a variety of delivery methods (e.g., quizzes, encouragement of critical thinking, team learning tasks). Their content focused on the concepts of gaming and gambling, erroneous beliefs/misconceptions about gambling attitudes towards gambling and money, sensation seeking and problem gambling. In every session, the researchers emphasised the establishment of a safe space to learn, which is recognised as an important aspect of effective learning with adolescents (Ayub et al., 2022). This study assessed gambling behaviour via questions relating to the amount of time spent gambling in the last week, and money gambled. Other outcomes included the Questionnaire of Misconceptions and Knowledge About Gambling (Ferland et al., 2002), DSM-IV-Multiple Response-Juvenile (DSM-IV-J-MR; Fisher, 2000), Attitudes Towards Gambling Scale (ATGS8; Wardle et al., 2011), and the Brief Sensation Seeking Scale (BSSS; Hoyle et al., 2002). Data about changes in thoughts, attitudes and behaviour, collected both immediately post-intervention and at 6 weeks, indicated that the programme was successful in increasing knowledge of gambling harms but did not identify specific factors contributing to efficacy. However, results relating to changes in gambling behaviour were unclear – although participants reported less time spent gambling post-intervention, there was no change in how much money they spent and no change in sensation-seeking behaviours.

More recently, Grahler et al. (2024) investigated the effectiveness of an app-based intervention in Germany (Pietsch et al., 2023) designed to encourage vocational students to voluntarily reduce or abstain from a self-selected addictive behaviour, including substance use, gambling, and gaming, over a two-week period. The intervention introduced the concept of habits and risky health behaviours and provided an explanatory video, guiding participants in using the app and selecting a personalised behavioural-change challenge. Participants received daily push notifications to assess their confidence in achieving their goal and to report on their progress. Upon completion, they could download and share a certificate. The study employed baseline assessments, 30-day follow-up self-reports, and app usage data from the intervention group, including frequency of app use, challenge selection, and personal relevance. The outcome variable measured health-promoting changes in the past-month, with dichotomous outcomes (change vs no change) for gambling behaviours. Results indicated that students who selected gambling-related challenges and engaged actively with the app showed a meaningful reduction in gambling.

#### Theme 2: Effective Interventions are Theory-Driven and Use Trained Practitioners

Theories associated with positive change in knowledge or behaviour include; the Dual-Process Theory and Conceptual Change Model, biopsychosocial models of gambling that incorporate behaviour-analytic perspectives, the Extended Parallel-Process Model and the Action Research Model, harm-minimisation models, and cognitive behavioural theories of self-efficacy (Broussard & Wulfert, 2017; Celio & Lisman, 2014; Donati et al., 2018, 2022; Martens et al., 2015; Neighbors et al., 2015; Parham et al., 2019; Tani et al., 2021; Turner et al., 2008b). André et al. (2022) used a Relapse Prevention Intervention based around CBT theories, whereas Neighbors et al. (2015) built their intervention strategy around social identity theory. Both interventions saw changes in reported gambling behaviour (albeit small effects by André et al., 2022), when assessed via the NODS-CLiP and SOGS six months post-intervention. Due to the wide range of theories and variety of studies, it is beyond the scope of this review to determine which theories were most effective.

Some studies used the Dual-Process theory of cognitive processing (which describes the interaction between intuitive and deliberate thought), through mathematical skills training that aimed to reduce misconceptions and cognitive distortions about gambling, addressing concepts such as the ‘mindware gap’ (not understanding rationality, probability and logic) or the ‘gambler’s fallacy’ (mistaken beliefs about randomness) associated with gambling harms (Frey & Neys, 2022; Goodie et al., 2019; Keen et al., 2019), in order to encourage correct probability skills and illusions of control. In addressing these fallacies, the studies aimed to improve analytical thinking and reduce harmful gambling behaviour (Armstrong et al., 2020); however, research in this area seems inadequate and solid conclusions are problematic (Delfabbro et al., 2006).

Several studies (Donati et al., 2014, 2018;, 2022; Ren et al., 2019; Taylor & Hillyard, 2009; Turner et al., 2008a, 2008b) focused on improving mathematical skills training that was reported in past research (Petry et al., 2017) in relation to CYP populations. These studies reported effectiveness in improving mathematical cognitions. However, efficacy in reducing reported gambling behaviour was mixed, with some studies suggesting a reduction (Donati et al., 2014, 2018, 2022), some having no effect (Turner al., 2008a), and others not assessing gambling behaviour (Turner et al., 2008b).

Among studies that examined school-based interventions, some trained the teachers who delivered the intervention (Walther et al., 2013) to boost its potential in reaching students. Two Italian studies demonstrated that training teachers to deliver support reduced gambling behaviour and increased gambling knowledge in children post-intervention (Donati et al., 2022; Tani et al., 2021). These outcomes were assessed through the Gambling Related Knowledge Scale (GRKS-A; Donati et al., 2019) the Gambling Behaviour Scale for Adolescents (Primi et al., 2015), the Gambling Related Cognitions Scale in Italian (Iliceto et al., 2015) and the SOGS-RA (Winters et al., 1993). The authors suggest that the ongoing influence of the teacher training on future students will bring about additional benefits (Tani et al., 2021). However, at this stage, it is not possible to determine the long-term effects of any of the interventions described in these articles due to lack of follow-up. Although the involvement of trained professionals can enhance intervention outcomes, scalability remains a significant challenge. Research by Grahler et al. (2024) has highlighted the potential of digital tools to complement professional-led interventions (Pietsch et al., 2023), thereby increasing their scalability without diminishing effectiveness. Future approaches may benefit from integrating professional expertise with digital innovations to develop more comprehensive and accessible gambling prevention strategies for diverse populations.

#### Theme 3: The Developmental and Individual Appropriateness of Interventions

Some studies that adapted interventions according to developmental age were found to be effective. For example, Ren et al. (2019) incorporated specific changes in a PowerPoint presentation depending on whether the participants were in primary, middle or high school. The presentation involved slides that covered the nature of gambling, misconceptions about gambling, teaching about randomness, probability and house advantage, rational and irrational beliefs and how to spot problem gambling. The material for younger audiences did not include detail relating to mathematical probability, whilst the content for the oldest participants included more complex material. Although this study did not assess results by age, a previous study, using the same intervention (Taylor & Hillyard, 2009) reported that the primary school audience (ages 8–11 years old) displayed the most improvement in pre-post-test scores short-term.

Parham et al. (2019), in the USA, considered the cultural relevance of their material to an urban, low socioeconomic status (SES), predominantly African American target population in Maryland. When designing and implementing the intervention, the authors considered the population’s needs by using feedback from previous implementations of the programme to address issues with terminology and simplifying the language to reduce barriers related to low reading/maths skills. Significant increases in student awareness and knowledge of gambling were reported following participation. Additionally, focus group data collected from programme facilitators suggested high student engagement and participation, programme feasibility, and ease of implementation. These results highlight the effectiveness of tailoring interventions to specific population needs.

The findings of Grahler et al. (2024) further underscore the importance of developmental and individual relevance of interventions. The app-based approach used by Pietsch et al. (2023) demonstrated greater efficacy when participants engaged with challenges aligned with their personal habits. This aligns with broader research emphasising the necessity of interventions that account for the unique contexts and behaviours of target populations to maximise effectiveness.

A study conducted with 13–16-year-olds in Canada aimed to highlight the consequences of harmful gambling behaviour for relationships, psychological health, and emotional health. The intervention included watching a 25-min docudrama featuring testimony of someone experiencing problem gambling, followed by real-life scenarios that illustrated the impact of adolescent gambling on relationships and mental health (St-Pierre et al., 2017). However, this study led to more positive attitudes towards gambling among young people.

#### Theme 4: Effective Interventions Involve Engagement with Digital Technologies

This theme addresses how some studies used technologies (e.g., smartphones, online games, app-based interventions, and a simulated gambling accelerator game) to actively engage participants in interventions (Broussard & Wulfert, 2017; Canale et al., 2016; Grahler et al., 2024; Lupu & Lupu, 2013; McAfee, 2020; McGivern et al., 2019; Pietsch et al., 2023; Todirita & Lupu, 2013; Zhou et al., 2019). These studies reported positive behaviour changes in the participants, including greater awareness about gambling fallacies and knowledge, fewer intentions to gamble, increased knowledge, and significant improvement in ‘erroneous attitudes and cognitions’, illusions of control and misconceptions, or reduction in gambling activities (Canale et al., 2016; Grahler et al., 2024; Lupu & Lupu, 2013; Pietsch et al., 2023; Todirita & Lupu, 2013; Zhou et al., 2019). However, as demonstrated by Grahler et al. (2024) and Pietsch et al. (2023), digital technologies can effectively support individuals who are willing to modify a behaviour.

## Discussion

This scoping review addressed two primary objectives: evaluating the range of existing interventions targeting CYP at risk of gambling-related harm and assessing the effectiveness of these interventions in supporting CYP with respect to gambling. Additionally, we aimed to identify research gaps that warrant further investigation. We identified 40 studies conducted between 2008 and 2025. In relation to the first objective, interventions we reviewed included psychological, educational (skills training and prevention) approaches, social norms and public health strategies. While some studies focused on changing gambling behaviour, others aimed at correcting misconceptions or increasing knowledge. Considering the type of intervention (see Table 2), there were 21 universal interventions (applicable to all youth), six indicated interventions (for CYP who display psychological or behavioural signs of gambling disorder but do not meet diagnostic criteria), 12 selective interventions (for CYP who share a characteristic which is known to increase risk of gambling-related harm) and only one treatment intervention (targeting CYP with a diagnosable gambling disorder). Many studies were school based, hence effectively reaching large numbers of CYP. These studies utilised multiple forms of didactic media such as lectures, discussions, role play, and docudrama to enhance CYP’s engagement and receptiveness. Additionally, many interventions aimed to improve other developmentally important skills such as mathematical ability, reasoning, and emotion regulation, as well as targeting gambling-related harms. Many interventions were grounded in sound theoretical frameworks, ensuring their relevance and effectiveness. Furthermore, numerous studies prioritised the acceptability of interventions to CYP, thereby increasing their potential impact and uptake.

In relation to the second objective, there were four main findings. First, interventions that include multiple methods and involve active engagement of participants were more effective in raising awareness and addressing negative attitudes about gambling. Second, effective interventions were theory-driven and used trained practitioners. Third, effective interventions were developmentally appropriate, adapted to individual characteristics, relevant to the target population, and often harnessed family support networks. Fourth, interventions that involved engagement with digital technologies led to positive behaviour change, which manifested in greater awareness about gambling, fewer intentions to gamble, and reductions in gambling activities.

Interventions that integrated multiple methods and actively engaged participants showed the most promise. Interventions combining educational components, skill development, and psychological approaches were particularly effective in raising awareness and altering attitudes toward gambling. For instance, educational interventions corrected misconceptions about gambling and led to increased knowledge, while psychological interventions, such as CBT, targeted behavioural changes. School-based interventions, especially those involving teacher training, emerged as effective strategies, not only reducing gambling behaviour but also increasing gambling-related knowledge among students (e.g., Donati et al., 2022). The success of these programmes underscores the potential of leveraging existing educational infrastructures to deliver preventive interventions. On the other hand, sample populations were mainly from educational settings such as schools, colleges, and universities, which raises concerns about the applicability of findings to CYP outside these settings, such as those not in formal education or in alternative education systems. Only one study (André et al., 2022) recruited participants from a child and adolescent psychiatry service, highlighting a significant gap in understanding how gambling interventions could support CYP already engaged with mental health services.

Effective interventions were developmentally appropriate and relevant to the target population (e.g., Taylor & Hillyard, 2009). Studies tailoring their content to participants’ developmental needs were often more effective, suggesting that a one-size-fits-all approach is insufficient for diverse age groups. Findings from research demonstrating the importance of considering the cultural relevance of material to a specific population (Parham et al., 2019) align with research emphasising the necessity for more inclusive interventions (Bailie et al., 2023; Castro et al., 2004), which advocates for the incorporation of populations often overlooked in research such as individuals with language, physical, social, or developmental differences. On the other hand, interventions that have demonstrated effectiveness in adults, such as lived experience facilitation (e.g., Thomas et al., 2023) may be counterproductive for young people, increasing positive attitudes to gambling (e.g., St-Pierre et al., 2017). However, the wide age range of participants in most of the reviewed studies meant the results cannot be applied to specific age groups, and more research on the impact of developmentally informed adaptation is needed, based on specific groups. Although research acknowledges that gambling may begin as early as 10–11 years (Emond & Griffiths, 2020; Forrest & McHale, 2012), only three interventions included younger children aged 8–10 years (Ren et al., 2019; Turner et al., 2008a; Walther et al., 2013). Although Turner et al. (2008a) used a programme designed for children under 18 years, they did not provide information on the participants’ average age or the ways in which it was adapted for younger participants. This oversight highlights the necessity of detailing how interventions are tailored to specific age groups. Interventions should consider the unique motivations and developmental needs of children and adolescents (Bronfenbrenner, 1986; Yeager et al., 2018).

Digital technologies played a significant role in several interventions, with studies reporting positive behavioural changes among participants. Smartphones, online games, app-based interventions, and simulated gambling environments likely facilitated engagement and convenience, making these interventions appealing to young people. This is in line with research that digital game interventions for mental health treatment and promotion are well received by young people based on positive outcomes, user satisfaction and high retention rates (Ferrari et al., 2020; Vié et al., 2024). However, it is unclear why incorporating digital technologies was effective. Future research should delve deeper into the mechanisms behind their success. Furthermore, few studies used digital technologies, especially for younger children and adolescents. Understanding these mechanisms could enhance the design and implementation of digital interventions, ensuring they are not only engaging but also substantively effective.

Additionally, our scoping review identified various research gaps in the populations targeted. Limited literature explored effective interventions for CYP with additional needs or who experience co-occurring mental health issues or alcohol addiction, and no studies explicitly included children and adolescents with Special Educational Needs and Disabilities (SEND). Additional targeted research aimed at CYP with SEND is needed, as evidence (Breyer et al., 2009; Chamberlain et al., 2023; Faregh & Derevensky, 2011; Geidne et al., 2016; Taylor et al., 2015) suggests that individuals with SEND (e.g., learning difficulties, attention deficit/hyperactivity disorder, autism) are more vulnerable to gambling-related harms than the general population. SEND-specific interventions therefore need to be developed. More generally, diverse populations should be included when implementing and evaluating the effectiveness of existing interventions. Additionally, no studies evaluated interventions involving family members, Gypsy/Roma/Traveller children, those with mental health issues, caregiving responsibilities, or homeschooled children, nor did any studies consider motivations for gambling. The broader social environment must be considered by including parents and carers in educational workshops and public health communications, involving those outside mainstream schools, and overcoming barriers related to language and content to ensure accessibility and user-friendliness (Liverpool et al., 2020; Silvers et al., 2019).

Several methodological weaknesses were identified across the studies. First, attrition rates were underreported. This can significantly skew results and undermine the validity of findings (Crutzen et al., 2015). Second, long-term follow-up assessments, which are crucial for determining the sustained impact of interventions, were lacking. Without such data, it is challenging to ascertain whether observed behavioural changes are temporary or indicative of lasting change. Third, in many studies, questionnaires designed for use with adults were adapted for use with children. Adult scales may not be applicable or appropriate for child populations (Bell, 2007), and older scales may not be useful in the modern world, given significant changes in gambling practices in recent years. Additionally, although self-report measures represent an appropriate way to measure change in beliefs, they do not necessarily represent actual change in beliefs, as participants may wish to present socially desirable viewpoints (Schell et al., 2021) or may be subject to recall bias (Althubaiti, 2016). Grahler et al. (2024) tackled this issue by implementing an app-based intervention (Pietsch et al., 2023) that gathered both behavioural and self-reported data, providing a more comprehensive assessment of changes in gambling and other risky behaviours. This approach exemplifies the potential of integrating digital tools to improve the accuracy and depth of intervention evaluations.

Most interventions focused on individual rather than socio-environmental factors. We recommend that researchers examine the role of reciprocal interactions between CYP and their immediate and wider environments. Current interventions are typically developed by adults without incorporating CYPs’ perspectives. To enhance intervention effectiveness, we recommend involving CYP in the development process, considering their perceptions, experiences, and communication methods. The highly adaptive aspects of the adolescent period allow for a greater capacity for resilience, positive risk-taking, development of cognitive skills, and autonomy. For example, asset-based interventions may consider focusing on young people’s skills, rather than their vulnerability. Social sensitivity could be framed as a skill to prevent gambling harms, rather than a vulnerability to them. Acknowledging the positive attributes of youth, such as their digital expertise and openness to new experiences, can lead to the creation of more acceptable interventions, potentially improving engagement and retention rates.

Additionally, a focus on knowledge and mathematical skills training assumes an individual responsibility to avoid gambling addiction, without consideration of wider aspects of responsibility and risk. This focus on individual behaviour places the responsibility for change on the individual rather than examining the role of adults in changing the environment to protect the child. As a result, some researchers recommend that treatment should aim to address not only individual factors but also the social contexts and environments involved in gambling behaviour, as some programmes are currently doing (Voll et al., 2022). Additionally, risk factors for taking up gambling may differ from protective factors that facilitate quitting among those who started previously. As such, interventions for individuals already displaying gambling-related harms should be designed differently from those focusing on prevention. This distinction was observed in the reviewed studies, where preventive interventions were mainly psycho-educational or educationally based, whereas treatment interventions used harm-minimisation strategies, psychological techniques, and social-norms approaches.

The studies reviewed indicate that most interventions targeting gambling-related harms among CYP are delivered as traditional educational programmes. Relevant and engaging interventions need to be implemented, as making interventions appealing to CYP increases their acceptability (Ferrari et al., 2020; Vié et al., 2024). This may involve interactive digital delivery modes, such as online games and simulated gambling activities, or involvement of media personalities, and cross-disciplinary partnerships that combine gambling expertise with social media expertise. Additionally, creating a safe and respectful environment is crucial for fostering meaningful engagement.

## Conclusions

This scoping review highlighted several key findings related to interventions aimed at mitigating gambling-related harm among CYP. The studies reviewed generally demonstrated high methodological quality with robust randomisation processes and clear reporting. However, most interventions targeted CYP in educational settings, which may limit the generalisability of the findings to other groups, such as those not in formal education or those engaged with mental health services. Additionally, while interventions varied widely in approach, from psychological and educational methods to public health strategies, those integrating multiple methods and actively engaging participants showed the most promise. The use of digital technologies also emerged as a significant trend, yet there is a critical need for deeper exploration into the mechanisms behind their effectiveness.

Despite its contributions, this review has several weaknesses that need to be addressed in future research. First, the reliance on academic databases may have resulted in omission of relevant unpublished studies, potentially skewing the findings. Evaluation of grey literature is recommended in future reviews. Second, the broad inclusion criteria, while capturing a wide range of interventions, may have introduced variability that complicated the synthesis of results. Additionally, due to the diverse range of studies, it was not possible to conduct a meta-analysis, meaning that the review cannot provide definitive conclusions about the effectiveness of different intervention strategies. Finally, the review focused on English-language publications, so might have excluded pertinent research in other languages, thereby limiting the comprehensiveness of the findings. Studies were predominantly conducted in North America and Europe, with a significant concentration in the USA, Canada, and Italy. This distribution underscores the global recognition of gambling as a public health issue, particularly in countries like the UK and Australia, where it has become a dominant concern over the past decade (Christopher, 2021).

## Supplementary Information

Below is the link to the electronic supplementary material.

Supplementary file1 (DOCX 27.5 KB)

## Data Availability

Our manuscript has no associated data.

## Footnotes

1 Demand-reduction interventions are designed to decrease the urge to gamble and prevent or limit the onset of problematic gambling. ^2^ Supply-reduction interventions are designed to restrict or interrupt the provision of gambling opportunities. ^3^ Interventions that target the gambling context may include removal of large note acceptors, maximum bets etc.
