# Supplementary material for: Mitigating Gambling-Related Harms in Children and Young People: A Scoping Review of Interventions
Source: J Gambl Stud. 2025 May 19;41(2):515–66. doi: 10.1007/s10899-025-10387-x (PMC12116823; doi:10.1007/s10899-025-10387-x)
Supplement: Supplementary file 1 — Supplementary file1 (DOCX 27.5 KB) [file 10899_2025_10387_MOESM1_ESM.docx]

## **Supplementary Materials: CASP^^[[1]](#footnote-1)^^ Checklists for the Study**

### CASP Randomised Controlled Trial Checklist

| ***20 STUDIES IN TOTAL*** | | **RANDOMISED CONTROLLED TRIALS** | | | | | | | | | | | | | | | | | | | |
| --- | --- | --- | --- | --- | --- | --- | --- | --- | --- | --- | --- | --- | --- | --- | --- | --- | --- | --- | --- | --- | --- |
|  |  | 1 | 2 | 3 | 4 | 5 | 6 | 7 | 8 | 9 | 10 | 11 | 12 | 13 | 14 | 15 | 16 | 17 | 18 | 19 | 20 |
|  |  | Pietsch et al (2023) | Primi et al (2022) | McAfee et al (2020) | Calado et al (2020) | Zhou et al (2019) | Donati et al (2018) | Broussard & Wulfert (2017) | St-Pierre et al (2017) | Canale et al (2016) | Dixon et al (2016) | Neighbors et al (2015) | Martens et al (2015) | Donati et al (2014) | Celio & Lisman (2014) | Walther et al (2013) | Larimer et al (2013) | Lupu & Lupu (2013) | Todirita & Lupu (2013) | Petry et al (2009) | Turner et al (2008a) |
| **A** | **IS STUDY DESIGN VALID?** | | | | | | | | | | | | | | | | | | | | |
| 1 | **Did the study address a clearly focused research question?** | **yes** | **yes** | **yes** | **yes** | **yes** | **yes** | **yes** | **yes** | **yes** | **yes** | **yes** | **yes** | **yes** | **yes** | **yes** | **yes** | **yes** | **yes** | **yes** | **yes** |
| 2 | **Was the assignment of participants to interventions randomised?** | **yes** | **yes** | **yes** | **yes** | **yes** | **yes** | **yes** | **yes** | **yes** | **yes** | **yes** | **yes** | **yes** | **yes** | **yes** | **yes** | **yes** | **yes** | **yes** | **no** |
| 3 | **Were all participants accounted for in it's inclusion?** | **yes** | **can't tell** | **yes** | **yes** | **can't tell** | **can't tell** | **yes** | **yes** | **can't tell** | **can't tell** | **yes** | **yes** | **yes** | **no** | **yes** | **yes** | **yes** | **can't tell** | **yes** | **can't tell** |
| **B** | **WAS THE STUDY METHODOLOGICALLY SOUND?** | | | | | | | | | | | | | | | | | | | | |
| 4 | **Were participants blind to intervention?** | **yes** | **no** | **can't tell** | **no** | **yes** | **no** | **yes** | **no** | **no** | **yes** | **yes** | **yes** | **no** | **yes** | **no** | **yes** | **yes** | **can't tell** | **yes** | **no** |
| 5 | **Were the study groups similar at the start of the study?** | **yes** | **yes** | **yes** | **yes** | **can't tell** | **yes** | **can't tell** | **yes** | **yes** | **yes** | **no** | **yes** | **yes** | **yes** | **yes** | **yes** | **yes** | **yes** | **yes** | **yes** |
| 6 | **Apart from intervention, did each study group receive the same level of care?** | **yes** | **yes** | **yes** | **yes** | **yes** | **yes** | **yes** | **yes** | **yes** | **yes** | **yes** | **yes** | **yes** | **yes** | **yes** | **yes** | **yes** | **yes** | **yes** | **yes** |
| **C** | **WHAT ARE THE RESULTS?** | | | | | | | | | | | | | | | | | | | | |
| 7 | **Were the effects reported comprehensively?** | **yes** | **yes** | **yes** | **yes** | **yes** | **yes** | **yes** | **yes** | **yes** | **yes** | **yes** | **yes** | **yes** | **yes** | **yes** | **yes** | **yes** | **yes** | **yes** | **no** |
| 8 | **Was the precision of the estimate of the intervention or treatment reported?** | **yes** | **yes** | **yes** | **can't tell** | **can't tell** | **yes** | **yes** | **yes** | **yes** | **yes** | **yes** | **yes** | **yes** | **yes** | **yes** | **yes** | **yes** | **yes** | **yes** | **yes** |
| 9 | **Do the benefits outweigh the harms?** | **yes** | **yes** | **no** | **no/can't tell** | **no/can't tell** | **yes** | **yes** | **no** | **no** | **yes** | **yes** | **yes** | **can't tell** | **yes** | **yes** | **yes** | **yes** | **yes** | **yes** | **yes** |
| **D** | **WILL THE RESULTS HELP LOCALLY?** | | | | | | | | | | | | | | | | | | | | |
| 10 | **Can the results be applied to your local population or context?** | **yes** | **yes** | **can't tell** | **can't tell** | **can't tell** | **can't tell** | **can't tell** | **can't tell** | **can't tell** | **can't tell** | **can't tell** | **can't tell** | **can't tell** | **can't tell** | **can't tell** | **can't tell** | **can't tell** | **can't tell** | **can't tell** | **yes** |
| 11 | **Would the experimental intervention provide greater value to the people in your care than any of the existing interventions?** | **yes** | **yes** | **can't tell** | **can't tell** | **can't tell** | **can't tell** | **can't tell** | **no** | **can't tell** | **can't tell** | **can't tell** | **can't tell** | **can't tell** | **can't tell** | **can't tell** | **can't tell** | **can't tell** | **can't tell** | **can't tell** | **can't tell** |

## CASP Cohort Study Checklist

| *7 STUDIES IN TOTAL* | | CROSS-SECTIONAL STUDIES | | | | | LONGITUDINAL STUDIES | |
| --- | --- | --- | --- | --- | --- | --- | --- | --- |
|  |  | Hansen & Rossow (2010) | Latvala et al (2022) | Nordmyr & Österman (2016) | Raisamo et al (2015) | Rossow et al (2013) | Ren et al (2019) | Tani et al (2021) |
| **A** | **ARE THE RESULTS OF THE STUDY VALID?** | | | | | | | |
|  | Did the study address a clearly focused issue? | yes | yes | yes | yes | yes | yes | yes |
|  | Was the cohort recruited in an acceptable way? | yes | yes | yes | yes | yes | yes | yes |
|  | Was the exposure accurately measured to minimise bias? | yes | yes | yes | yes | yes | yes | yes |
|  | Was the outcome accurately measured to minimise bias? | yes | yes | yes | yes | yes | yes | yes |
|  | Have the authors identified all important confounding factors? | can't tell | yes | yes | yes | yes | yes | yes |
|  | Have they taken account of the confounding factors in the design and/or analysis? | yes | yes | yes | yes | yes | yes | yes |
|  | Was the follow up of subjects complete enough? | yes | yes | yes | yes | yes | yes | yes |
|  | Was the follow up of subjects long enough? | yes | yes | yes | yes | yes | yes | yes |
| **B** | **WHAT ARE THE RESULTS?** | | | | | | | |
|  | What are the results of this study? | positive | positive | positive | positive | positive | positive | positive |
|  | Do you believe the results? | yes | yes | yes | yes | yes | yes | yes |
| **C** | **WILL THE RESULTS HELP LOCALLY?** | | | | | | | |
|  | Can the results be applied to the local population? | possibly | possibly | possibly | possibly | possibly | yes | yes |
|  | Do the results of this study fit with other available evidence? | yes | yes | yes | yes | yes | yes | yes |
|  | What are the implications of this study for practice? | restrictions can help as part of an overall ecological outlook | more research on changing nature of adolescent gambling needed | low prevalence of gamblers |  | authors state that youth may still use other forms of unregulated gambling |  | look into training teachers |

### CASP Checklist for Remaining Studies

| ***13 STUDIES IN TOTAL*** | | **EXPERIMENTAL PRE-POST STUDIES WITH 1 GROUP** | | | | | | **EXPERIMENTAL PRE-POST STUDIES WITH 2 GROUPS** | | | | **PILOT STUDIES** | | **EXPLORATORY STUDY** |
| --- | --- | --- | --- | --- | --- | --- | --- | --- | --- | --- | --- | --- | --- | --- |
|  |  | Grahler et al. (2024) | Chóliz et al (2022) | Dodig Hundric et al (2021) | Donati et al (2022) | Parham et al (2019) | Taylor & Hillyard (2009) | Huic et al (2017) | Turner et al (2008b) | Williams et al (2010) | Wohl et al (2013) | André et al (2022) | McGivern et al (2019) | Diehr et al (2018) |
| **A** | **IS THE STUDY DESIGN VALID?** | | | | | | | | | | | | | |
| 1 | **Did the study address a clearly focused research question?** | **yes** | **yes** | **yes** | **yes** | **yes** | **yes** | **yes** | **yes** | **yes** | **yes** | **yes** | **yes** | **yes** |
| 2 | **Was the assignment of participants to interventions randomised?** | **yes** |  |  |  |  |  |  |  | **yes** |  | **yes** | **yes** |  |
| 3 | **Were all participants accounted for in its inclusion?** | **can't tell** | **can't tell** | **no** | **yes** | **yes** | **can't tell** | **can't tell** | **yes** | **yes** | **yes** | **yes** | **no** |  |
| **B** | **WAS THE STUDY METHODOLOGICALLY SOUND?** | | | | | | | | | | | | | |
| 4 | **Were participants blind to intervention?** |  |  |  |  |  |  | **no** | **no** | **no** | **yes** | **no** | **can't tell** |  |
| 5 | **Were the study groups similar at the start of the study?** |  |  |  |  |  |  | **yes** | **yes** | **yes** | **yes** | **can't tell** | **can't tell** |  |
| 6 | **Apart from intervention, did each study group receive the same level of care?** |  |  |  |  |  |  | **yes** | **yes** | **yes** | **yes** | **yes** | **yes** |  |
| **C** | **WHAT ARE THE RESULTS?** | | | | | | | | | | | | | |
| 7 | **Were the effects reported comprehensively?** | **yes** | **yes** | **yes** | **yes** | **can't tell** | **yes** | **yes** | **yes** | **yes** | **yes** | **yes** | **yes** | **yes** |
| 8 | **Was the precision of the estimate of the intervention or treatment reported?** | **yes** | **can't tell** | **yes** | **yes** | **no** | **yes** | **yes** | **yes** | **yes** | **yes** | **yes** | **yes** | **no** |
| 9 | **Do the benefits outweigh the harms?** | **can't tell** | **yes** | **can't tell** | **yes** | **can't tell** | **yes** | **yes** | **yes** | **yes** | **can't tell** | **yes** | **can't tell** | **yes** |
| **D** | **WILL THE RESULTS HELP LOCALLY?** | | | | | | | | | | | | | |
| 10 | **Can the results be applied to your local population or context?** | **yes** | **yes** | **can't tell** | **yes** | **can't tell** | **yes** | **yes** | **yes** | **yes** | **no** | **possibly** | **no** | **no** |
| 11 | **Would the experimental intervention provide greater value to the people in your care than any of the existing interventions?** | **yes** |  | **can't tell** | **yes** | **can't tell** | **can't tell** | **can' tell** | **can't tell** | **can't tell** | **no** | **yes** | **no** | **can't tell** |

1. CASP stands for Critical Appraisal Skills Programme. CASP offers critical appraisal skills checklist tools that are used to systematically assess the trustworthiness, relevance and results of published papers. CASP checklist tools are designed for use with Systematic Reviews, Randomised Controlled Trials, Cohort Studies, Case Control Studies, Economic Evaluations, Diagnostic Studies, Qualitative studies and Clinical Prediction Rule. [↑](#footnote-ref-1)
